# The diagnostic value of ^68^Ga-NOTA-MAL-Cys-MZHER_2:342_ PET/CT imaging for HER2-positive lung adenocarcinoma

**DOI:** 10.3389/fmed.2024.1447500

**Published:** 2024-08-13

**Authors:** Shu Li, Ke Wang, Xue Zhu, Donghui Pan, Ling Wang, Xu Guo, Xiaomin Gao, Qing Luo, Xun Wang

**Affiliations:** ^1^Department of Pulmonary and Critical Care Medicine, Jiangnan University Medical Center, Jiangnan University (Wuxi No. 2 People's Hospital), Wuxi, Jiangsu, China; ^2^Wuxi School of Medicine, Jiangnan University, Wuxi, Jiangsu, China; ^3^NHC Key Laboratory of Nuclear Medicine, Jiangsu Key Laboratory of Molecular Nuclear Medicine, Jiangsu Institute of Nuclear Medicine, Wuxi, Jiangsu, China; ^4^Wuxi Maternity and Child Health Care Hospital, Affiliated Women's Hospital of Jiangnan University, Wuxi, Jiangsu, China; ^5^Nantong University Medical School, Nantong, Jiangsu, China

**Keywords:** ^68^Ga-NOTA-MAL-Cys-MZHER2:342, ^18^F-FDG, PET, lung cancer, HER2

## Abstract

**Background:**

The human epidermal growth factor receptor 2 gene (HER2) has been identified as a potential therapeutic target in lung adenocarcinoma (LUAD). Non-invasive positron emission tomography (PET) imaging provides a reliable strategy for *in vivo* determination of HER2 expression through whole-body detection of abnormalities. The PET tracer ^68^Ga-NOTA-MAL-Cys-MZHER_2:342_ has shown promising results for HER2-positive breast and gastric cancers. This study aims to evaluate the performance of ^68^Ga-NOTA-MAL-Cys-MZHER_2:342_*in vitro* and *in vivo* models and in clinical patients with HER2-positive LUAD.

**Methods:**

NOTA-MAL-Cys-MZHER_2:342_ was synthesized and labeled with ^68^Ga. Cell uptake, cell binding ability, and stability studies of ^68^Ga-NOTA-MAL-Cys-MZHER_2:342_ were assessed both in the Calu-3 lung cancer (LC) cell line and normal mice. *In vivo* assessment in tumor-bearing mice was conducted using microPET imaging and biodistribution experiments. Additionally, preliminary PET/CT imaging analysis was performed on HER2-positive LC patients.

**Results:**

^68^Ga-NOTA-MAL-Cys-MZHER_2:342_ was prepared with a radiochemical purity (RCP) exceeding 95%. The tracer demonstrated high cell uptake in HER2-overexpressing Calu-3 cells, with an IC_50_ of 158.9, an adequate 1.73 nM. Good stability was exhibited both *in vitro* and *in vivo*. MicroPET imaging of Calu-3-bearing mice revealed high tumor uptake and notable tumor-to-background ratios. Positive outcomes were also observed in two HER2-positive LUAD patients.

**Conclusion:**

^68^Ga-NOTA-MAL-Cys-MZHER_2:342_ demonstrated satisfactory stability, sensitivity, and specificity. These findings suggest that ^68^Ga-NOTA-MAL-Cys-MZHER_2:342_ PET/CT imaging provides a novel tool for non-invasive visual assessment of HER2 expression in LUAD patients.

## Introduction

LC has emerged as the leading cause of malignant tumor-related mortality worldwide, accounting for 1.8 million deaths annually (1–3). Adenocarcinoma, comprising approximately 40% of all LC cases, represents the most prevalent subtype. The implementation of molecular targeted therapy has significantly improved the overall prognosis and quality of life for patients with LUAD. However, the absence of effective targeted agents for numerous driver mutations underscores the necessity for novel therapeutic approaches. Among these genetic alterations, human epidermal growth factor receptor 2 (HER2/ERBB2) mutations are observed in 2–3% of LUAD cases (4, 5). HER2 alterations, encompassing mutations, amplifications, and overexpression, are associated with aggressive tumor growth and elevated metastasis rates. Furthermore, acquired HER2 amplification has been proposed as a mechanism of resistance to EGFR/ALK tyrosine kinase inhibitors (TKIs), further substantiating its role in tumorigenesis (6, 7). Recent years have witnessed the emergence of novel compounds, such as trastuzumab deruxtecan (T-DXd, DS-8201) and poziotinib, which have increased the objective response rate (ORR) to approximately 50% for patients with HER2-positive LUAD (8, 9). Consequently, the detection of HER2 status in LUAD has become crucial for developing and selecting appropriate treatment regimens.

Current diagnostic methods for HER2-positive cancer primarily rely on fluorescence *in situ* hybridization (FISH) and immunohistochemistry (IHC) (10). While these techniques yield relatively clear results for tumors with absent or overexpressed HER2, accurately quantifying HER2 in tumors with moderate expression remains challenging (11). PET, a three-dimensional molecular imaging modality utilizing radioisotopes, has demonstrated significant clinical value in cancer diagnosis (12, 13). ^18^F-fluorodeoxyglucose positron emission tomography-computed tomography (^18^F-FDG PET/CT) is currently the most widely employed tracer in clinical practice. However, this approach primarily assesses tumor metabolic activity rather than detecting the status of driver genes (14, 15). To develop a non-invasive method for predicting which cancer patients will benefit from HER2-targeted therapy, extensive research has focused on developing PET tracers targeting this receptor. ^64^Cu-DOTA-trastuzumab, derived from trastuzumab, has shown promising results *in vitro* and *in vivo* for detecting HER2-positive lung and breast cancers (16, 17). ^99m^Tc-Z_HER2:V2_-pemetrexed enables precise visualization and treatment in a HER2-positive (A549) xenograft model through the combination of HER2 affinity and pemetrexed (18, 19). Moreover, the ZHER_2:342_ Affibody conjugated with N-[2-(4-^18^F-fluorobenzamido)ethyl]maleimide has demonstrated specificity in evaluating HER2 expression *in vivo* and in breast cancer metastasis models (20, 21). However, complex synthesis procedures, lower RCP, and prolonged targeting times have limited their clinical application. Previous research has demonstrated that the hydrophilic linker (GGGRDN)-modified ZHER_2:342_ affibody (MZHER_2:342_), when coupled with maleimide-NOTA (MAL-NOTA), yields a conjugate that exhibits notable tumor-to-background ratios following labeling with ^18^FAl/^68^Ga (22–25). To date, clinical studies on HER2-targeted tracers in LUAD remain scarce. The present study aims to investigate the efficacy of the HER2 affibody tracer ^68^Ga-NOTA-MAL-Cys-MZHER_2:342_ for PET imaging of HER2-positive LUAD and compare its performance with that of ^18^F-FDG.

## Materials and methods

### General materials

Fluorodeoxyglucose (FDG) was supplied by Wuxi Jiangyuan Industrial Technology and Trade Corporation, China. Cys-MZHER_2:342_ was synthesized by Shanghai Apeptide Biotechnology Corporation, China. NOTA-MAL-Cys-MZHER_2:342_ was prepared following established methods, achieving a chemical purity exceeding 95% (25). The ^68^Ge/^68^Ga generator was procured from Isotope Technologies Garching (ITG), Germany. All other reagents utilized were of analytical grade.

### Preparation of ^68^Ga-NOTA-MAL-Cys-MZHER_2:342_

NOTA-MAL-Cys-MZHER_2:342_ (150 μg, 25 nmol) was dissolved in 30 μL of deionized water. Subsequently, ^68^GaCl_3_ eluent (185 MBq, 2 mL) and 1 M sodium acetate solution (120 μL) were added, maintaining a pH range of 3–3.5. The resulting mixture was heated in an oil bath at 70°C for 10 min. Following heating, the mixture was diluted with 8 mL of deionized water and transferred to an activated BOND ELUT C18 column (Varian Medical Systems, United States). Impurities removed by washing with 10 mL of deionized water. The product was then eluted using 0.3 mL of 10 mM HCl ethanol. The eluent was further diluted with 5 mL of saline and filtered through a 0.22 μm Millipore filter into a sterile vial. RCP was assessed using high-performance liquid chromatography (HPLC, Waters, United States) in accordance with previously established protocols (24, 26).

### Cell lines and culture

The human LC cell lines Calu-3 and NCI-H520 were obtained from the Cell Bank of Shanghai Institutes for Biological Sciences, China. These cell lines were cultured in RPMI-1640 medium supplemented with 10% fetal bovine serum (FBS, Gibco Life Technologies, United States) and 1% penicillin–streptomycin (P-S) solution. The culture conditions were maintained at 37°C in a humidified atmosphere containing 5% CO_2_.

### Cell uptake and block studies

For the cell uptake assay, Calu-3 (HER2-positive) and NCI-H520 (HER2-negative) cells were seeded in 12-well plates at a density of 1 × 10^6^ cells per well and cultured overnight. After washing with PBS, the cells were incubated with 370 KBq of ^68^Ga-NOTA-MAL-Cys-MZHER_2:342_ at 37°C in a 5% CO_2_ environment for 15, 30, 60, and 120 min, respectively. In the cell block study, sufficient quantities of unlabeled Cys-MZHER_2:342_ (1 μM) were added to the 12-well plates and incubated for 10 min prior to re-incubation with ^68^Ga-NOTA-MAL-Cys-MZHER_2:342_ for various durations. Following incubation, the cells were washed with ice-cold PBS and lysed with 1 mL of NaOH (1 M) for 1 min. The lysate was transferred to γ-counting tubes and measured using a γ counter (Perkin Elmer Instruments Corporation, United States). Cell uptake was expressed as a percentage of decay-corrected radiation dose per counting tube (%AD/10^5^ cells).

### Cell binding assay

For the cell binding assay, Calu-3 cells were seeded in 24-well plates at a density of 1 × 10^5^ cells per well and cultured overnight. After washing with PBS, the cells were incubated with 370 KBq of ^68^Ga-NOTA-MAL-Cys-MZHER_2:342_ in conjunction with varying concentrations of unlabeled Cys-MZHER_2:342_ peptide. Following a 2-h incubation period, the cells were washed with ice-cold PBS and lysed by adding 1 M NaOH for 1 min. The resulting lysate was transferred to γ-counting tubes and measured using a γ counter. The 50% inhibitory concentration (IC_50_) value was calculated using GraphPad Prism 8.0 software (San Diego, United States).

### Stability analysis

The stability of ^68^Ga-NOTA-MAL-Cys-MZHER_2:342_ was evaluated through *in vitro* and *in vivo* experiments. For *in vitro* stability, 3.7 MBq of the radiotracer was incubated with FBS or phosphate-buffered saline (PBS) at 37°C. The RCP was analyzed by HPLC at 15, 30, 60, and 120 min. *In vivo* stability was assessed in normal male mice (4 weeks old, Changzhou Cavins Laboratory Animals Ltd., China). These mice were administered 37 MBq of ^68^Ga-NOTA-MAL-Cys-MZHER_2:342_ via tail vein injection. Blood samples were collected in heparinized centrifuge tubes at 15-, 30-, and 60-min post-injection. The collected blood was immediately centrifuged at 10,000 rpm for 3 min. Protein precipitation was achieved by adding 500 μL of acetonitrile to the resulting plasma. The mixture was subsequently centrifuged at 9,000 rpm for 5 min to obtain the supernatant. The supernatant was filtered through a microporous membrane and subjected to HPLC analysis.

### *In vivo* microPET imaging

Male BALB/c nude mice (4 weeks old, Changzhou Cavins Laboratory Animals Ltd., China) were utilized for *in vivo* microPET imaging. Calu-3 cells (5 × 10^5^) were subcutaneously injected into these mice. Subsequent experiments were conducted when the tumor size reached approximately 300 cubic millimeters (10 days post-injection). For imaging, mice received a tail vein injection of 100 μL of ^68^Ga-NOTA-MAL-Cys-MZHER_2:342_ (3.7 MBq). Static PET images were acquired for 10 min at 1-h post-injection (*n* = 4 per group). Blocking experiments were performed by co-injecting an excess amount of unlabeled Cys-MZHER_2:342_ with the labeled ^68^Ga-NOTA-MAL-Cys-MZHER_2:342_ into Calu-3-bearing mice. A 10-min static PET scan was conducted 1-h post-injection. Quantitative analysis of PET images was performed according to previously reported methods (27). Animal experiments were approved by the Animal Research Committee of Jiangsu Institute of Atomic Medicine (JSINM-2022-061).

### *Ex vivo* biodistribution analysis

Mice bearing Calu-3 tumors were injected with approximately 740 KBq of radiolabeled ^68^Ga-NOTA-MAL-Cys-MZHER_2:342_. These mice were euthanized at predetermined time points. Blood, tumors, and major organs were harvested and weighed. Radioactivity measurements were conducted using a gamma counter. The data were calculated and expressed as a percentage of the injected dose per gram of tissue (%ID/g).

### Clinical patients and PET imaging

This clinical study was approved by the Ethics Committee of the Peking University Cancer Hospital (NCT04547309). This study included two LUAD patients without severe hepatic or renal dysfunction. All patients provided written informed consent. PET/CT scans were performed using a Biograph 64 PET/CT scanner (Siemens Medical Solutions, Nuremberg, Germany). Following the injection of 74 MBq of ^68^Ga-NOTA-MAL-Cys-MZHER_2:342_, patients were positioned supine on the scanning bed. Dynamic scans were conducted for 60 min, covering the brain and whole-body regions. PET images were reconstructed using three-dimensional ordered-subset expectation maximization. Biodistribution analysis was performed using PET images. Regions of interest (ROIs) for the tumor and major organs (e.g., brain, lung, heart, liver, spleen, and kidney) were delineated with the assistance of corresponding CT images. Standardized uptake values (SUVs) were calculated according to previously described methods (22, 28).

### Histology and immunohistochemistry

Tumor tissues were surgically excised from each mouse and allowed to decay radioactively for over 48 h to ensure sufficiently reduced radioactivity levels. Subsequently, the tissues were fixed in formalin, embedded in paraffin, and sectioned. For immunohistochemical staining, the sections were incubated with HER2 antibody (ab134182, Rabbit mAb, Abcam, United States) overnight at 4°C, followed by a 2-h incubation with an HRP-conjugated secondary antibody at room temperature. Visualization of the sections was achieved using a DAB kit (Beyotime, China), and observations were made using a light microscope (Olympus IX53, Tokyo, Japan). HER2 expression levels were classified into four grades (0+ to 3+) based on the number and intensity of tumor cell membrane staining. HER2 expression in the tumor tissue was categorized as either positive (IHC: 2+ to 3+) or negative (IHC: 0+ to 1+).

### Statistical analysis

Statistical analyses were conducted using GraphPad Prism (v. 5.0, GraphPad software). Comparisons between groups were evaluated using the Student’s *t*-test and one-way analysis of variance (ANOVA). Statistical significance was defined as a *p*-value of <0.05.

## Results

### Chemistry and radiochemistry

**Figure 1:**
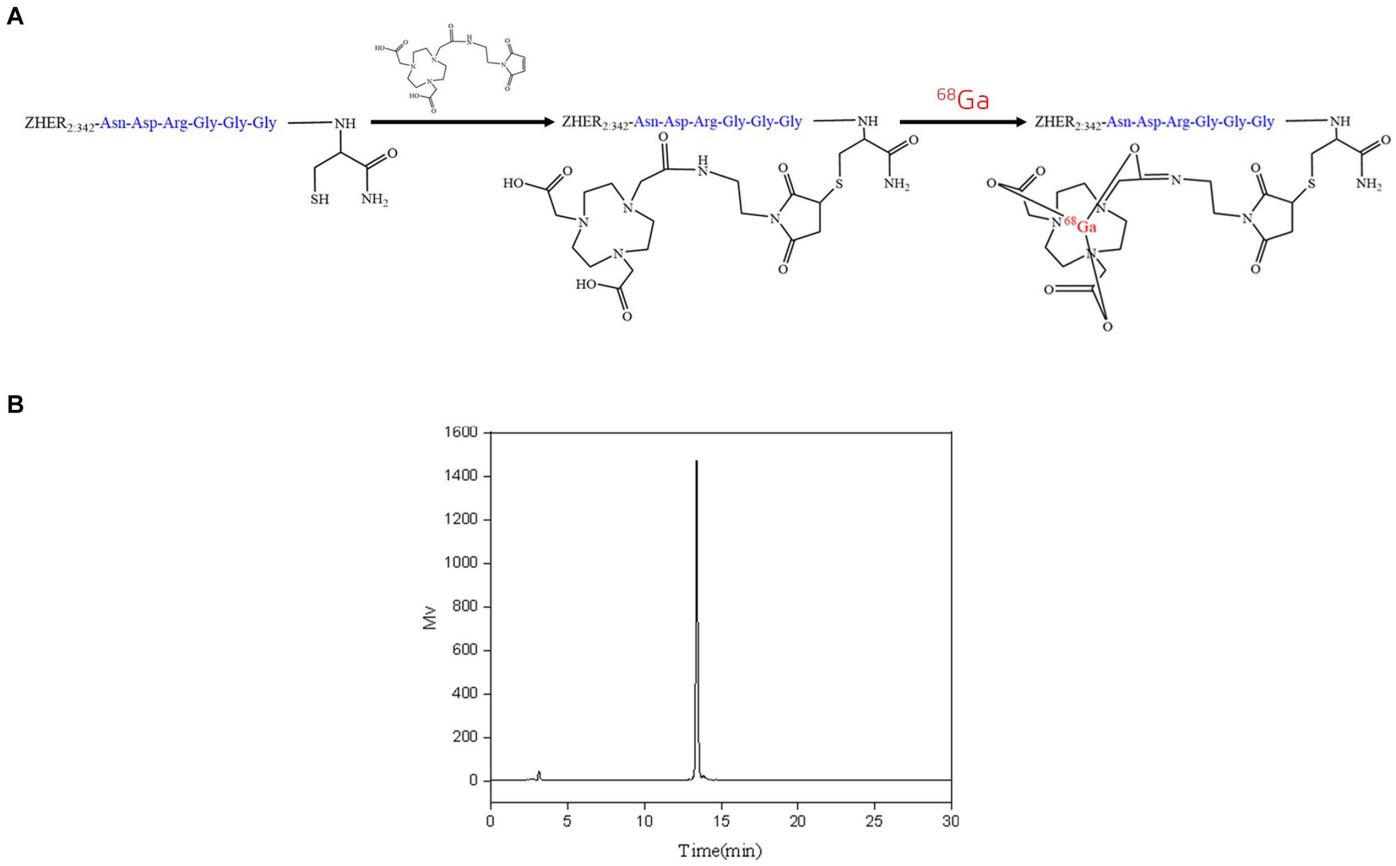
Radiolabeling of NOTA-MAL-Cys-MZHER_2:342_. **(A)** Synthetic methodology for ^68^Ga-NOTA-MAL-Cys-MZHER_2:342_. **(B)** High-performance liquid chromatography (HPLC) detection of ^68^Ga-NOTA-MAL-Cys-MZHER_2:342_ radioactivity.

The conjugation of NOTA-MAL with Cys-MZHER_2:342_ was performed at 40°C, resulting in the acquisition of NOTA-MAL-Cys-MZHER_2:342_ with an approximate yield of 50%. An efficient ^68^Ga labeling method was used for ^68^Ga-NOTA-MAL-Cys-MZHER_2:342_. The probes were produced within 30 min with a yield of 62.53 ± 5.24% (*n* = 3) (Figure 1A, based on ^68^Ga, non-decay-corrected). Analytical HPLC determined that the RCPs of the compounds exceeded 90% (Figure 1B).

### Cell uptake and binding assays and stability analysis

**Figure 2:**
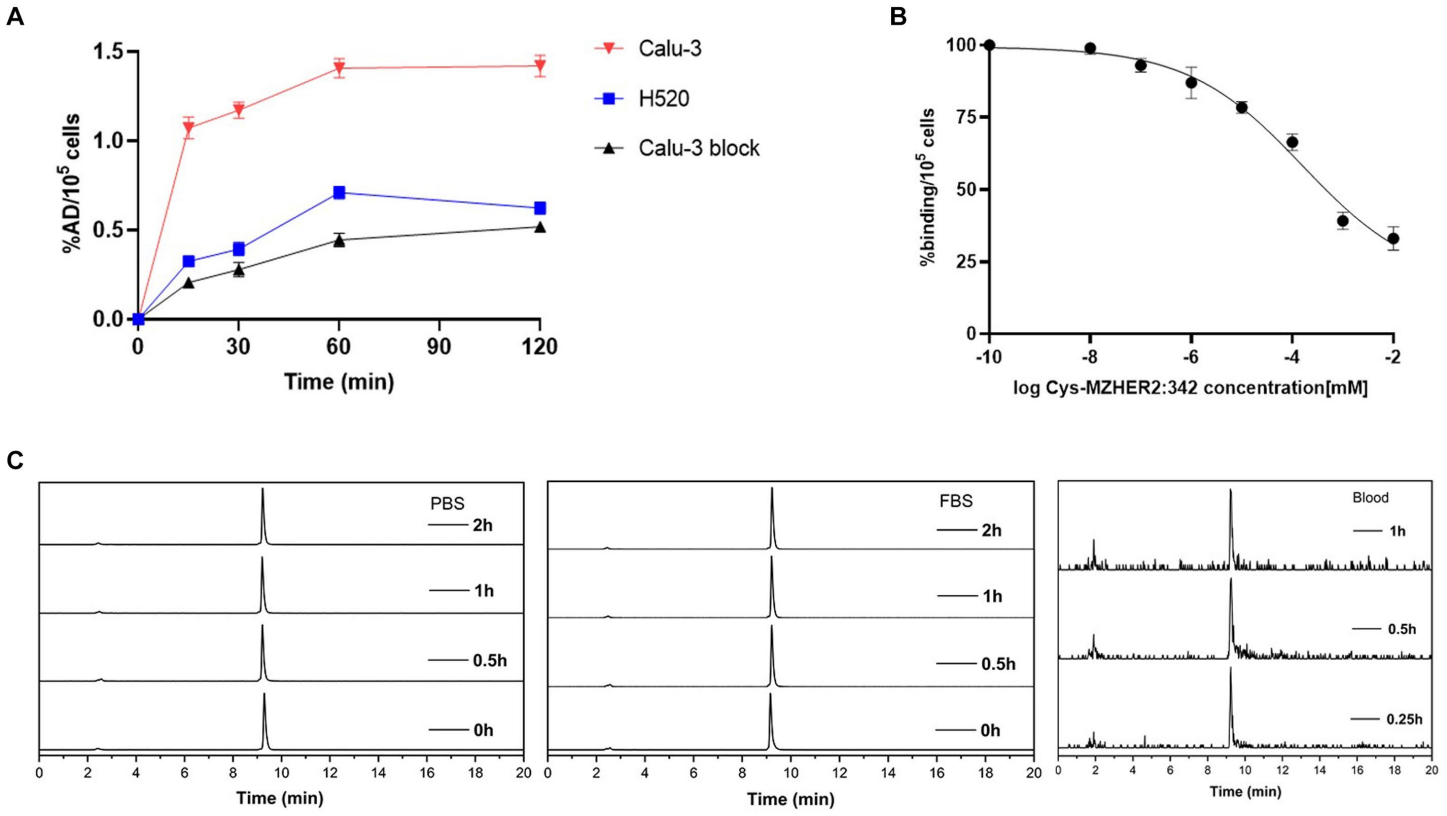
Cell uptake, cell binding, and stability of ^68^Ga-NOTA-MAL-Cys-MZHER_2:342_. **(A)** Cell uptake of ^68^Ga-NOTA-MAL-Cys-MZHER_2:342_ in Calu-3 and NCI-H520 cell lines. **(B)** Competitive cell binding assay of ^68^Ga-NOTA-MAL-Cys-MZHER_2:342_ in Calu-3 cells. **(C)** HPLC chromatographic profiles of ^68^Ga-NOTA-MAL-Cys-MZHER_2:342_ following *in vitro* and *in vivo* experimental procedures.

The cell uptake and block studies were conducted utilizing Calu-3 and NCI-H520 cell lines. As illustrated in Figure 2A, Calu-3 cells (HER2-positive) exhibited a cell uptake of 1.16 ± 0.01 %AD/10^5^ cells after 60 min of incubation. This uptake was significantly reduced to 0.44 ± 0.04 %AD/10^5^ cells (*n* = 3, *p* < 0.001) upon the addition of unlabeled Cys-MZHER_2:342_. Conversely, NCI-H520 cells (HER2-negative) demonstrated a cell uptake of 0.71 ± 0.04 %AD/10^5^ cells (*p* < 0.001), which was comparable to the results obtained in the cell block assay. A competitive binding assay was subsequently performed. Figure 2B demonstrates that unlabeled Cys-MZHER_2:342_ inhibited the binding of ^68^Ga-NOTA-MAL-Cys-MZHER_2:342_ to Calu-3 cells in a dose-dependent manner, with an IC_50_ of 158.9 ± 1.73 nM (*n* = 3). *In vitro* stability analysis revealed that ^68^Ga-NOTA-MAL-Cys-MZHER_2:342_ maintained stability at 37°C for a minimum of 2 h in both FBS and PBS solutions, retaining 95% purity after 2 h of incubation. Furthermore, the compound exhibited good stability 1-h post-injection *in vivo* (Figure 2C).

### MicroPET imaging and biodistribution studies

**Figure 3:**
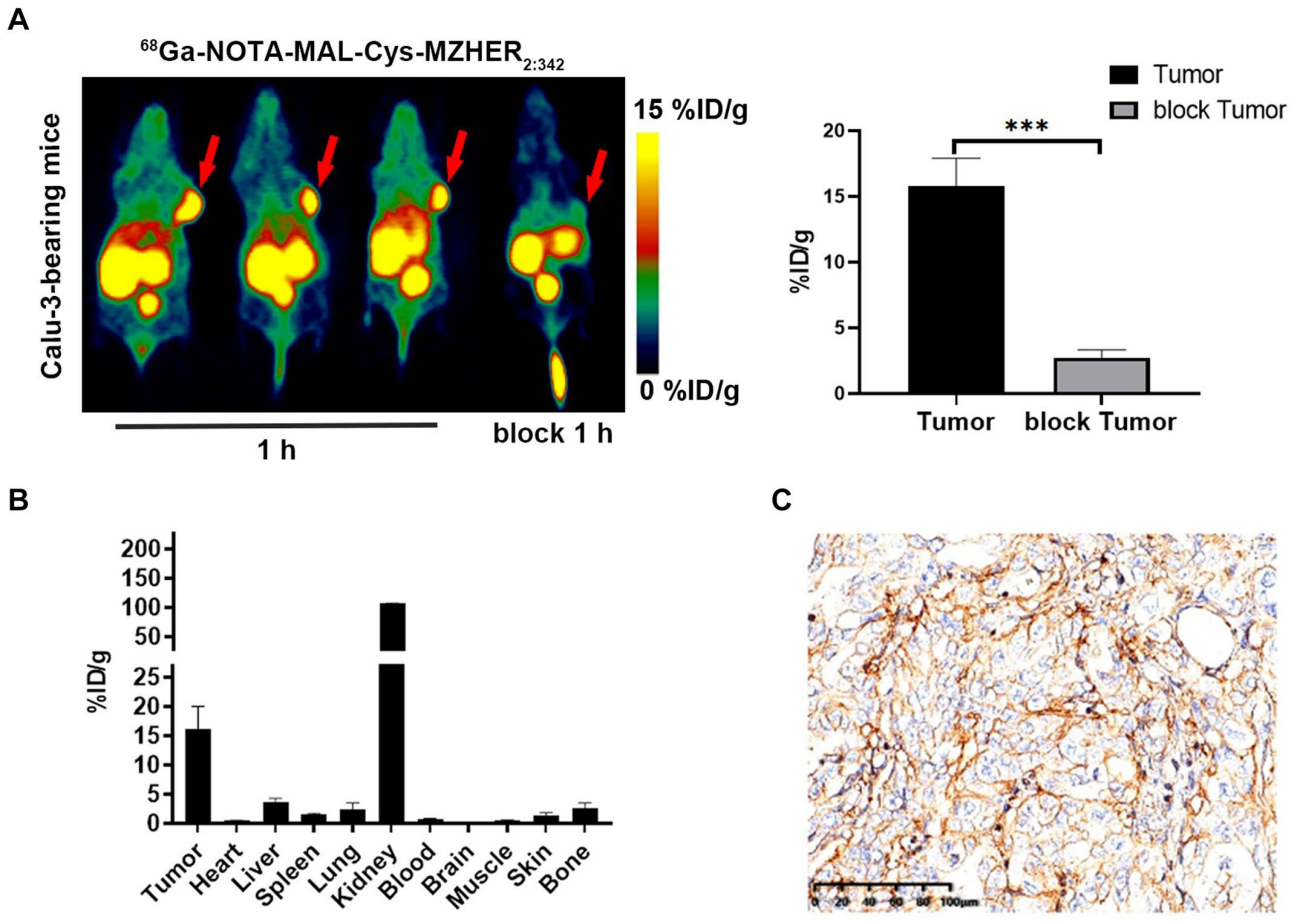
MicroPET imaging and biodistribution studies of ^68^Ga-NOTA-MAL-Cys-MZHER_2:342_ in a xenograft model. **(A)** Whole-body microPET images (*n* = 3) of HER2-positive Calu-3-bearing mice, including blocking experiments with excess unlabeled Cys-MZHER_2:342_. **(B)** Decay-corrected biodistribution of ^68^Ga-NOTA-MAL-Cys-MZHER_2:342_ in tumor, heart, liver, spleen, lung, kidney, blood, and muscle tissues (*n* = 3). **(C)** Immunohistochemical analysis (20× magnification) of tumor tissues using HER2-specific antibodies. ***p < 0.001.

Following radiolabeling, microPET scans were conducted to assess the efficacy of ^68^Ga-NOTA-MAL-Cys-MZHER_2:342_ in Calu-3-bearing mice. As depicted in Figure 3A, tumor uptake of ^68^Ga-NOTA-MAL-Cys-MZHER_2:342_ (15.77 ± 2.13 %ID/g) was observed 60-min post-injection, resulting in favorable tumor-background ratios. One hour after co-injection with Cys-MZHER_2:342_, the tumor signal was significantly reduced, with an uptake value of 2.73 ± 0.58 %ID/g. Notably, renal accumulation reached 103.4 ± 5.23 %ID/g at 1-h post-injection, substantially higher than in other normal tissues. This observation suggests that the tracer is primarily excreted through the renal system. Figure 3B and Table 1 illustrate the biodistribution of ^68^Ga-NOTA-MAL-Cys-MZHER_2:342_ in Calu-3-bearing mice. The tumor uptake was 16.11 ± 3.91 %ID/g, while uptake in the heart, liver, lung, and kidney was 0.5 ± 0.03, 3.55 ± 0.73, 2.44 ± 1.07, and 106.85 ± 0.61 %ID/g, respectively (*n* = 3). The tumor-to-blood and tumor-to-muscle uptake ratios were calculated to be 24.29 ± 1.93 and 35.77 ± 2.14, respectively. HER2 expression in tumor tissues was further evaluated through immunohistochemical assays, which revealed high expression levels (Figure 3C).

**Table 1:** Biodistribution of ^68^Ga-NOTA-MAL-Cys-MZHER_2:342_ in Calu-3-bearing mice.

Organ (%ID/g)	60 min
Tumor	16.11 ± 3.91
Heart	0.50 ± 0.03
Liver	3.55 ± 0.73
Spleen	1.47 ± 0.25
Lung	2.44 ± 1.07
Kidney	106.85 ± 0.61
Blood	0.67 ± 0.20
Brain	0.08 ± 0.02
Muscle	0.46 ± 0.14
Skin	1.43 ± 0.39
Bone	3.00 ± 0.84
**Ratios**	
Tumor/blood	24.29 ± 1.93
Tumor/muscle	35.77 ± 2.14

### PET imaging of clinical patients

**Figure 4:**
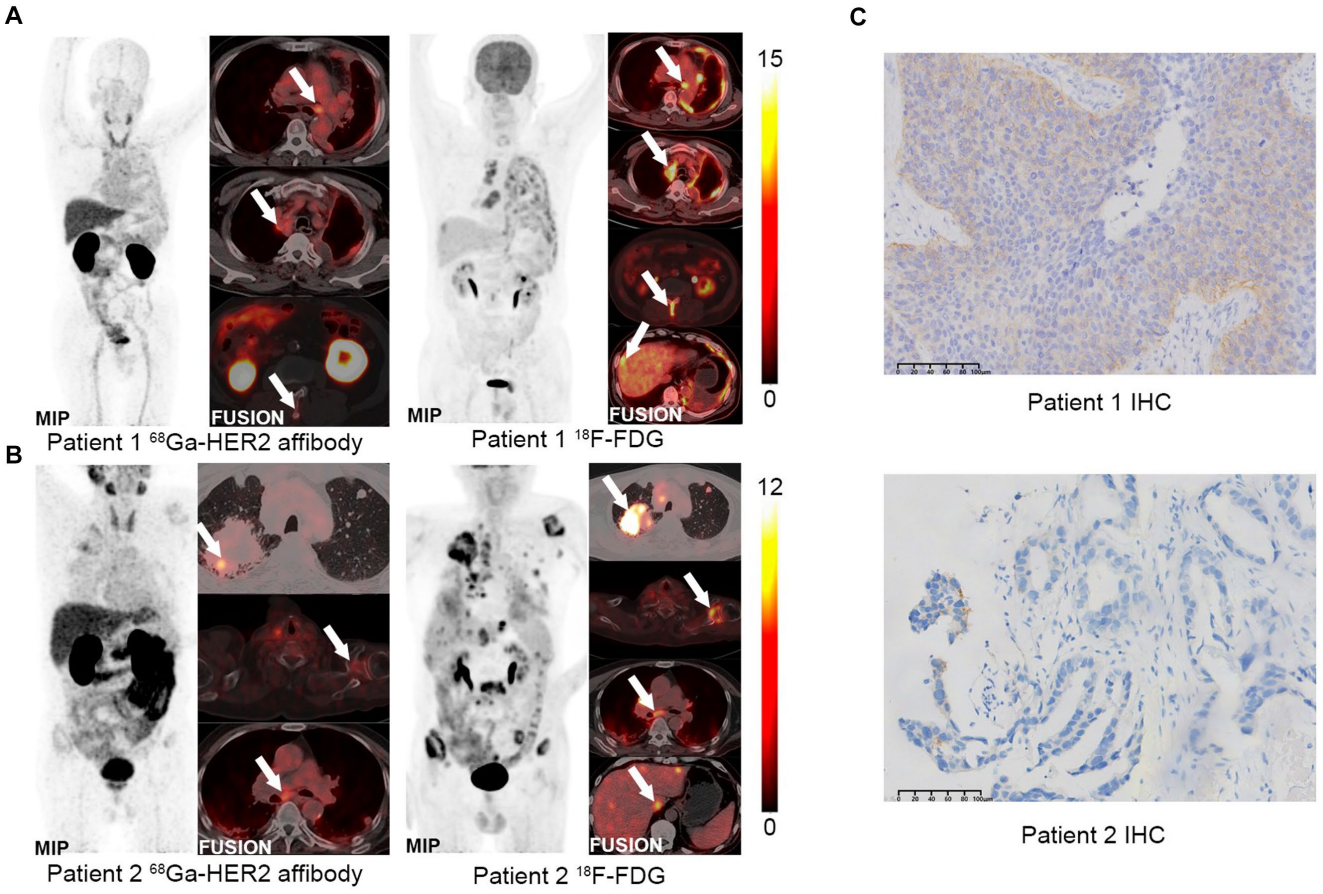
PET imaging of clinical patients using ^68^Ga-NOTA-MAL-Cys-MZHER_2:342_. **(A,B)** Whole-body PET images of HER2-positive LUAD patients using ^68^Ga-NOTA-MAL-Cys-MZHER_2:342_ and ^18^F-FDG, respectively. **(C)** Immunohistochemical analysis (20× magnification) of tumor tissues using HER2-specific antibodies.

PET/CT imaging was conducted on two HER2-positive LUAD patients using ^18^F-FDG and ^68^Ga-NOTA-MAL-Cys-MZHER_2:342_. As illustrated in Figure 4, both tracers exhibited uptake at tumor sites in the two patients, demonstrating favorable tumor-to-background ratios. Patient 1, 1-year post-primary resection for LUAD, displayed ^68^Ga-NOTA-MAL-Cys-MZHER_2:342_ SUVmax values of 9.6 ± 0.5, 6.0 ± 0.3, and 2.5 ± 0.1 in mediastinal lymph nodes, left pleura, and L2 spine, respectively. Comparatively, the corresponding ^18^F-FDG SUVmax values were 6.8 ± 0.2, 7.5 ± 0.3, and 6.3 ± 0.3. Concurrently, the liver exhibited an abnormal SUVmax value of 6.4 ± 0.4. In Patient 2, ^68^Ga-NOTA-MAL-Cys-MZHER_2:342_ PET/CT imaging revealed primary and multiple metastatic foci (Figure 4B). The SUVmax values for the right lung upper lobe, left scapula, and subcarinal lymph nodes (NO. 7) were 7.3 ± 0.3, 3.1 ± 0.2, and 4.9 ± 0.2, respectively. The corresponding ^18^F-FDG SUVmax values were 16.6 ± 0.7, 10.9 ± 0.5, and 6.6 ± 0.3. Immunohistochemical analysis was performed on biopsy samples obtained from the primary tumor tissue of both patients. As depicted in Figure 4C, patient 1’s biopsy sample scored HER2 3+, while patient 2 scored 1 + .

## Discussion

Affibody molecules, small engineered scaffold proteins comprising 58 amino acids, are extensively utilized for binding to target proteins. These molecules are employed in tumor diagnosis and therapy due to their high affinity for molecular recognition (29, 30). To date, a variety of radiolabeled HER2 affibody molecules have been developed to image HER2 in tumors. A HER2-binding affibody, ZHER_2:342_, has been screened and evaluated in preclinical and clinical PET studies (20, 23, 25). Different radioisotopes (^68^Ga, ^18^F) labeled ZHER_2:342_ and its analogs have demonstrated favorable performance in PET imaging. In our laboratory, ^68^Ga-NOTA-MAL-Cys-MZHER_2:342_ is synthesized and prepared within 30 min, maintaining high radiolysis purity without further purification. Preliminary studies have shown satisfactory sensitivity and specificity in PET imaging of HER2-positive breast and gastric cancers with good contrast (22, 23). The application of ^68^Ga-NOTA-MAL-Cys-MZHER_2:342_ to HER2-positive LC has not been evaluated until now. This study conducted evaluations of ^68^Ga-NOTA-MAL-Cys-MZHER_2:342_ *in vitro*, *in vivo*, and in clinical patients with HER2-positive LUAD.

Calu-3 cells, exhibiting high HER2 expression, were selected as positive cells, while NCI-H520 cells served as a negative control. In cell uptake experiments, NOTA-MAL-Cys-MZHER_2:342_ was labeled with ^68^Ga and cultured with cells for 2 h. The results demonstrated high uptake in Calu-3 cells and low uptake in NCI-H520 cells. The addition of unlabeled Cys-MZHER_2:342_ significantly decreased uptake in Calu-3 cells. The competitive binding assay revealed an IC_50_ of 158.9 ± 1.73 nM for this tracer, comparable to other HER2-targeting tracers (IC_50_ = 116.71 ± 1.28 nM) (25). The tracer also exhibited excellent stability under *in vitro* and *in vivo* conditions. In microPET imaging of xenograft models, ROI data indicated that ^68^Ga-NOTA-MAL-Cys-MZHER_2:342_ exhibited higher radioactivity levels in Calu-3 tumors than other healthy organs, except for the kidney. The uptake values were notably higher than those observed with ^99m^Tc-Z_HER2:V2-_pemetrexed (16.11 ± 3.91 %ID/g versus 2.6 ± 1.0 %ID/g) (18).

The diagnosis of tumor subtypes has traditionally relied on puncture biopsies, which are subject to high heterogeneity and dynamic expression, resulting in variable accuracy. Recent studies have demonstrated that misdiagnosis of HER2-positive breast cancer patients may occur when evaluating the efficacy of ADC drugs (31). Moreover, invasive puncture biopsies are not suitable for all patients. These limitations can be overcome by non-invasive PET/CT molecular imaging, which allows for the quantification of HER2 expression. While ^18^F-FDG, a glucose analog, plays a crucial role in detecting tissue metabolism, its utility in LC detection is limited. Studies have reported false positives in lymphoid follicles and pulmonary sclerosing hemangioma (32, 33). In contrast, HER2 receptor expression is typically minimal or absent in normal tissues. Compared to ^18^F-FDG, ^68^Ga-NOTA-MAL-Cys-MZHER_2:342_ provides a clearer visualization of HER2 expression in both primary and metastatic lesions, offering significant value in selecting individualized treatment regimens and assessing efficacy.

The *in vivo* results prompted further evaluation of ^68^Ga-NOTA-MAL-Cys-MZHER_2:342_ PET imaging in two patients with HER2-positive LUAD. The imaging demonstrated satisfactory tumor uptake and rapid clearance from normal organs, indicating high sensitivity and specificity in binding to HER2-positive LUAD cells. Notably, patient 2 exhibited negative IHC results; however, HER2 affibody PET/CT revealed high uptake, which was subsequently confirmed as HER2 amplification through genetic testing. This finding further underscores the importance of ^68^Ga-NOTA-MAL-Cys-MZHER_2:342_ in diagnosing HER2 expression levels in LC. This case emphasizes the potential of 68Ga-NOTA-MAL-Cys-MZHER_2:342_ as a valuable diagnostic tool, particularly in cases where traditional methods fail. The ability to identify HER2-positive lesions accurately can significantly impact treatment decisions, as patients with HER2-positive lesions are more likely to benefit from targeted therapies such as trastuzumab and other HER2 inhibitors. Furthermore, the use of PET/CT imaging can guide biopsy locations, ensuring that samples are taken from areas with the highest tracer uptake, thus improving the likelihood of detecting HER2-positive.

Despite its high sensitivity and specificity for HER2-positive LUAD, increased tracer accumulation was observed in the kidney and liver. This accumulation may present challenges in assessing patients with distant metastases. In the clinical evaluation of LC patients, the tracer was unable to detect metastases due to high renal metabolism. This contrasts with ^18^F-FDG, which successfully located corresponding areas of high metabolism (Figures 4A,B). Previous research has reported that HER2 affibody modified by an enzymolysis clearance strategy can effectively reduce renal uptake (34). This approach may offer a promising solution for reducing the non-tumor uptake of ^68^Ga-NOTA-MAL-Cys-MZHER_2:342_.

## Conclusion

In conclusion, ^68^Ga-NOTA-MAL-Cys-MZHER_2:342_ exhibits exceptional performance in both *in vitro* and *in vivo* models, as well as in clinical patients. This novel radiotracer holds significant potential for contributing to personalized clinical diagnosis and treatment strategies in the future.

## Data Availability

The raw data supporting the conclusions of this article will be made available by the authors, without undue reservation.
